# Editorial: Insights in neuroenergetics, nutrition and brain health: 2023

**DOI:** 10.3389/fnins.2023.1331872

**Published:** 2023-12-13

**Authors:** Avital Schurr, David J. Mokler, Hyoung-gon Lee, Carla I. Tasca

**Affiliations:** ^1^Department of Anesthesiology and Perioperative Medicine, School of Medicine, University of Louisville, Louisville, KY, United States; ^2^Department of Biomedical Sciences, College of Osteopathic Medicine, University of New England, Biddeford, ME, United States; ^3^Department of Neuroscience, Developmental and Regenerative Biology, The University of Texas at San Antonio, San Antonio, TX, United States; ^4^Laboratorio de Neuroquimica 4, Departamento de Bioquimica, Centro de Ciências Biológicas, Universidade Federal de Santa Catarina, Florianópolis, SC, Brazil

**Keywords:** brain health, Parkinson's disease, Moyamoya disease, migraine, intermittent energy restriction

This Research Topic issue comprises eight contributions, four original research papers, three review articles and one opinion paper. It is a relatively succinct Research Topic of articles that should provide a glimpse into the wide array of new insights into neuroenergetics, nutrition and brain health, an expanding subfield of neuroscience.

In their research article, Mubariz et al. described an investigation into the effect of mutations in the *GBA1* gene on the transcription factor EB (TFEB), the main regulator of the autophagy-lysosomal pathway. Their study demonstrated that the mammalian target of rapamycin complex 1 (mTORC1)-TFEB axis is a potential underlying mechanism for the pathogenesis of Parkinson's disease (PD). Yu et al. prospectively investigated the association between plasma urea cycle metabolites and the risk for the cerebrovascular condition known as Moyamoya disease (MMD), which is characterized by the progression of intracranial carotid artery stenosis and an abnormal vascular network in the brain. Their study indicated plasma urea cycle metabolites as potential biomarkers for the risk of MMD. Zhao et al. studied the possible association between selenium intake and migraine, the second most common neurological disorder, in the general American population. Intermittent energy restriction (IER) as an effective strategy of weight loss has gained popularity over the past decade. Li et al. investigated the effect of IER intervention on cerebral regional homogeneity (ReHo) using functional magnetic resonance imaging (fMRI) in 35 obese adults. Xue et al. evaluated the effectiveness of acupuncture as auxiliary treatment when combined with Western medicine therapeutics of epilepsy, reviewing the literature and performing a meta-analysis. Sun et al. reviewed the literature on the topic of mild cognitive impairment (MCI) and its association with liver disease via humoral factors derived from the gastrointestinal tract. They also examined the progress in magnetic resonance imaging (MRI) of the brain performed during MCI associated with liver disease. Zhang et al. examined the role of astrocytic metabolic pathways of carbohydrates, fatty acids and amino acids and changes in these metabolic pathways that offer perspectives on treatment and therapy for various neurological disorders such as depression, Alzheimer's disease and epilepsy. An opinion paper by Schurr uses the accumulated data in recent years to argue for the central role lactate plays in neuroenergetics. Overall, the collection of articles presented in this Research Topic provides insightful original data and review of knowledge on the importance of energy metabolism and nutrition on brain health. Future Research Topics will bring information about this unlimited field of study, contributing to the understanding of brain metabolism under physiological and pathological conditions.

## Author contributions

AS: Writing—original draft, Writing—review & editing. DM: Writing—review & editing. H-gL: Writing—review & editing. CT: Writing—review & editing.

